# Management of Comprehensive Care of multiple-birth infants from fetal to infancy period: challenges, training, strategies

**DOI:** 10.1186/s12887-021-02613-3

**Published:** 2021-03-29

**Authors:** Tahereh Changiz, Mahboobeh Namnabati

**Affiliations:** 1 Medical Education Research Center, Isfahan University of Medical Sciences, Isfahan, Iran; 2 Department of Pediatric and Neonates, Nursing and Midwifery Care Research Center, Faculty of Nursing and Midwifery, Isfahan University of Medical Sciences, Isfahan, Iran

**Keywords:** Care, COVID 19, Family, Infant, Multiple births, Premature

## Abstract

**Background:**

Prematurity escalates the crisis of the infants a susceptible group of the society. Multiple delivery further intensifies the susceptibility of both family and health system. A comprehensive care is, thus, necessary to ensure the optimal growth and development of such multiple-births. Accompanied by trainings, challenges, and strategies, the present study was conducted based on a two-year report of comprehensive care management experience on two sets of multiple infants.

**Methods:**

A qualitative case study approach was used to survey these two sets of premature infants (quadruplet and quintuplet) and their families. The data were collected through medical files, interviews, questionnaire, field presence, phone call and *WhatsApp* application, and continued follow-ups. Content analysis was performed based on survey and interventions during a period of two years in Isfahan, Iran (2018–2020).

**Results:**

Case presentation and comprehensive care management are the main areas resulted from this study. The results of the study were categorized in eight challenging areas (categories) and strategies including sterility and infertility period, transition from the intrauterine to neonatal intensive care unit (NICU), discharge process, physical and developmental status, home visit and home care, development of care plan, socio-economic support, and coronavirus nightmare.

**Conclusion:**

Based on challenges and strategies during these two years, the situation of the multiple-birth infants and their families’ needs should be identified as the first prerequisites in an inter-professional approach and in collaboration with the health providers. Isfahan University of Medical Sciences, Welfare Organization, and the charities were the parties involved with this process in our study. It was also found that developing a separate specific package of comprehensive care management plan for multiple-births is a necessity.

## Background

The risk of multifetal pregnancy and premature delivery has recently been increased through using advanced fertility technology, assisted reproductive technologies, and other fertility treatments. As a result, multifetal delivery has been increased 4 to 8 times in the united states during the last decade [1, 2]. In a 7-years prospective study conducted in Amin hospital in Isfahan, Iran, it was shown that 280 deliveries of total 18,790 deliveries were twins, of which 68% were same sex and 31.9% opposite sex (28.5% boys and %39.4 girls). Among the deliveries, 2% were majorly abnormal and 4.6% were with minor abnormality [3]. Another study conducted in Rasht, a city in north of Iran, indicated that 7% of the deliveries were twins [4].

A study was also conducted in a private hospital in Nigeria. Encompassing the high risk pregnancies, the study surveyed the outcomes and experiences related to multifetal deliveries from admission up to the discharge during a period of three years. The gestational age was 32 weeks and more than 80% of the infants suffered from respiratory distress syndrome, icteric, and species. The results of this study indicated that from 1950 deliveries, 0.72% were multifetal deliveries, 0.56% triplets, 0.1% quadruplets, and 0.05% quintuplets. There was no significant difference between survival rate of multiple births and that of the twins [5].

A 37-years-old first-time mother who was hospitalized from 23rd week of her pregnancy in Honolulu was the only quintuplet delivery reported. She delivered her quintuplets at 28th week and 6th day by cesarean and later was discharged with her infants in an acceptable condition [6].

Therefore, multifetal and preterm deliveries are not reduced, leaving the health teams with such infants. Although, the advancement of care and medical technology has increased the survival of such infants, their problems have remained intact [2]. According to Alz, “prematurity will remain with the family and the infant as a problem for the whole lifetime” [7]. A review of the studies conducted in Iran and other countries revealed that these infants will bear medical and psycho-social problems for the years ahead [8–10].

According to a review of the local newspapers and literature in Iran, there are numerous multiple-birth deliveries in different cities such as Tabriz, Hormozgan, Genaveh, Damavand, Mashhad, and Hamedan [11, 12]. The infants of a quadruplet, born in 2004, are now school children with many health difficulties like nutrition infection and re-hospitalization. Their family also has agonies and is in need of support and care [11]. The following is a case report of two sets of Iranian multifetal deliveries during 2018–2020. The needs and care management as well as the necessity of teamwork to implement home visits and cares of the deliveries were investigated in this study. It is believed that the survival of such infants can save the country’s capital and contribute to the population growth. However, health system can only contribute to the survival, growth, and development of preterm infants.

## Methods

### Study design

A qualitative study was conducted to survey the infants’ comprehensive care management plan deeply and accurately, enabling the researchers to review a complex phenomenon in a real field. In this approach, researchers explored the phenomenon within its context using a variety of data sources. A variety of lenses allows us to reveal and understand.

multiple facts of the phenomenon [13, 14]. Bearing in the mind that simultaneous involvement with the quintuplets and quadruplets is a very rear occasion, this approach was considered suitable for this study.

### Study setting and participants

The study was conducted in the houses of the quintuplet and quadruplet as the actual field of the study. Moreover, hospital, clinics, or physician offices were also used as alternative fields if necessary. The participants were the family members including father, mother, grandmother, aunt, and cousins. A quadruplet and a quintuplet and their parents (*n* = 13) were the main subjects of the study. In addition, physicians, nurses, speech and rehabilitation therapists, and benefactors who were interested in sharing their experiences and knowledge participated in the study.

### Data collection

The data were collected through questionnaire, interviews, participatory observation, field notes, and memos. Arranging it with the faculty authorities, the researchers and caregivers informed the parents about the aim of the study and care activities including interviews and taking and showing pictures to obtain the parents’ permission for entering their houses. The parents were also requested to proof the report of the given care and their consent.

### Data analysis

Content analysis was used for the interpretation of the content of textual data. This approach has been designed to describe different phenomena. The interviews were also transcribed and coded. Then, similar codes were grouped into categories. Many qualitative studies have incorporated this approach to the design and analysis of the study. Challenges were selected through categories of the study.

One of the best ways to increase the reliability of data is long-term involvement of the researcher with the research subjects. Thus, the researchers were most often interacting with the participants during the two years to ensure the reliability of data. Member checking was performed using expert informant. Dependability of findings was assured by data triangulation which involved the collection of data from multiple sources for the study.

## Results

**Fig. 1:**
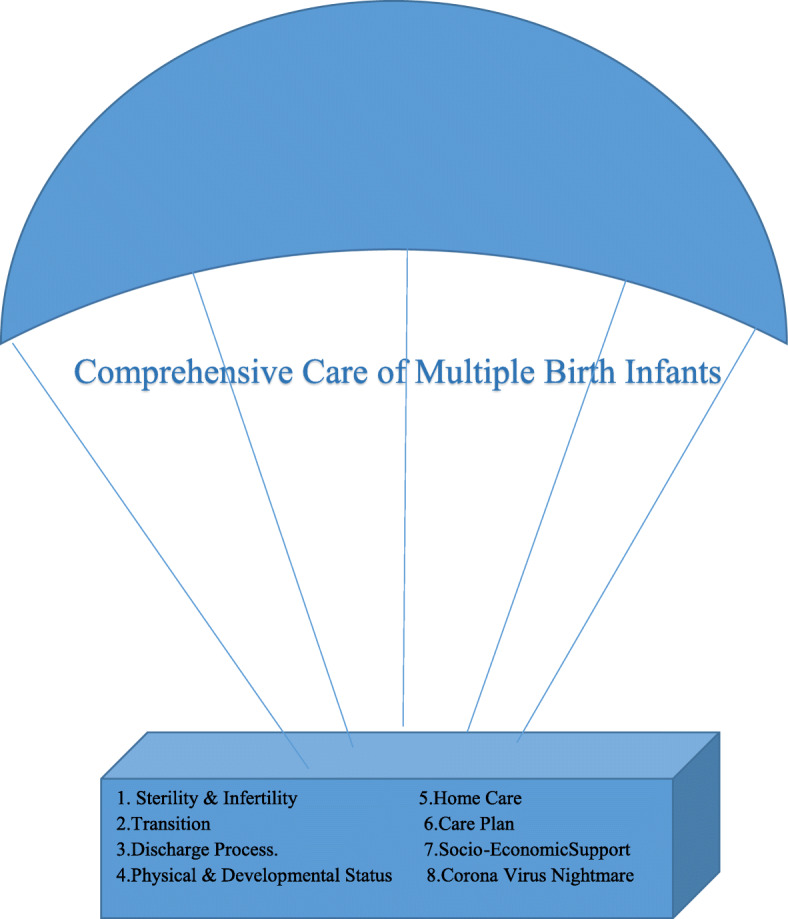
Comprehensive Care of Multiple Birth Infants (Namnabati, Isfahan University of Medical Sciences, Isfahan, Iran. 2020).

The results of the present study can be classified into eight challenging areas or categories including sterility and infertility period, transition from the intrauterine to NICU, discharge process of multiple births, physical and developmental status, home visit and home care, development of care plan, socio-economic support and COVID-19 nightmare (Fig. 1). Considerable points/strategies were proposed with regard to the challenging areas.

### Sterility and infertility period

Experiencing some problems like abortion and bleeding, most young couples are afraid of sterility and infertility, while they want to be parents soon after their marriage. Consequently, mothers are treated by assisted reproductive technology. Usually, the mothers who take inductive medications are young 24–26-years-old women. The mother of our quintuplet had experienced two abortions in her log and was under treatment with clomiphene tablets. At the same time the heart beats of five embryos could be heard through sonography. The mother was monitored by obstetrician during her pregnancy term, supported by her sister and her mother during 7 months of pregnancy at her mother’s home. She was hospitalized in 26th week of pregnancy and was medically administered by hydrocortisone, heparin, and other required interventions.

### Transition from intrauterine to NICU

Multiple-birth infants are normally hospitalized in the NICU for a few weeks up to two months to complete their gestational age. The *maternal placental* circulation in the fetal period and skin-to-skin contact were disconnected after birth and replaced by digital technology and the interactions of health providers. Other physiological needs of the infant including ventilation were met through medical and nursing cares. In the present study, the mother of the quintuplet said:

*“The infants were hospitalized in the NICU for 26 days and the physician told me that I did not need to stay in the ward any longer since they were connected to the equipment. So, I milked myself at home and brought it in bottles to the ward”.*

The nurses had used the milk to feed the infants. During the last days, the mother was allowed to enter the ward for dressing her infants. The fears of snuggling and milking as well as insufficient training about breathing interruptions, lack of required facilities at home, and re-hospitalization were the problems ahead of the families from the discharge moment up to coming months.

### Discharge process

The parents’ fear from the home care was the first challenge related to the discharge process. Their anxiety for not knowing what to do in case of apnea, feeding the infant if the oral/nasal gastric tube is removed during night and holidays when clinics are closed were added to the discharge problems that were escalated further by their financial shortcomings. Unemployed parents could not afford the charges of few weeks of hospitalization. Neither the limited social supports nor hospital discounts were enough for the parents to cover the charges by their low income, resulting in their further anxiety, lingering hospitalization and even more charges. Finally, the charge was compensated by the money donated by charities and the infants could be discharged. Therefore, the discharge itself is a challenge in the care management.

### Physical and developmental status

**Fig. 2:**
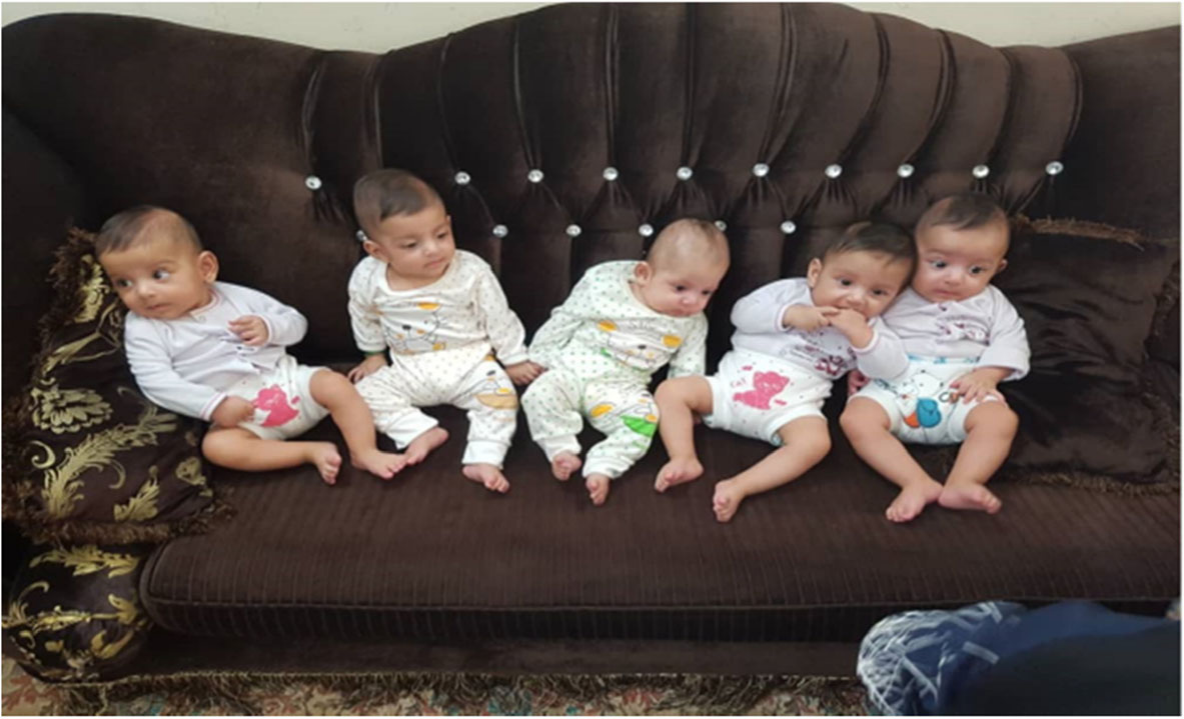
Picture 1: quintipelt at age of 8 months (Namnabati, Isfahan University of Medical Sciences, Isfahan, Iran.2020)

**Fig. 3:**
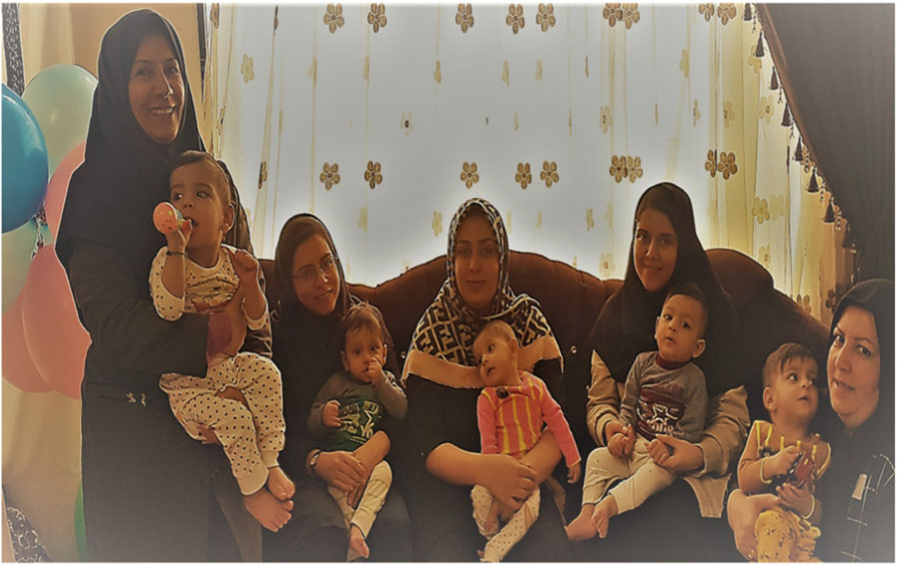
Picture 2: quintipelt at one year (Namnabati, Isfahan University of Medical Sciences, Isfahan, Iran.2020)

**Fig. 4:**
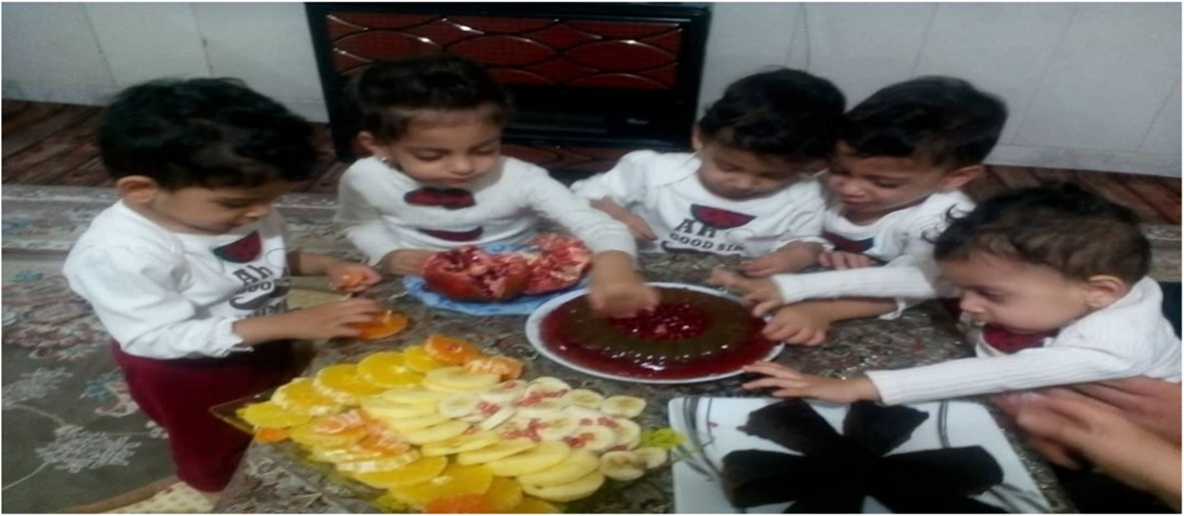
Picture 3: quintuplet at age of two (Namnabati, Isfahan University of Medical Sciences, Isfahan,Iran.2020)

**Fig. 5:**
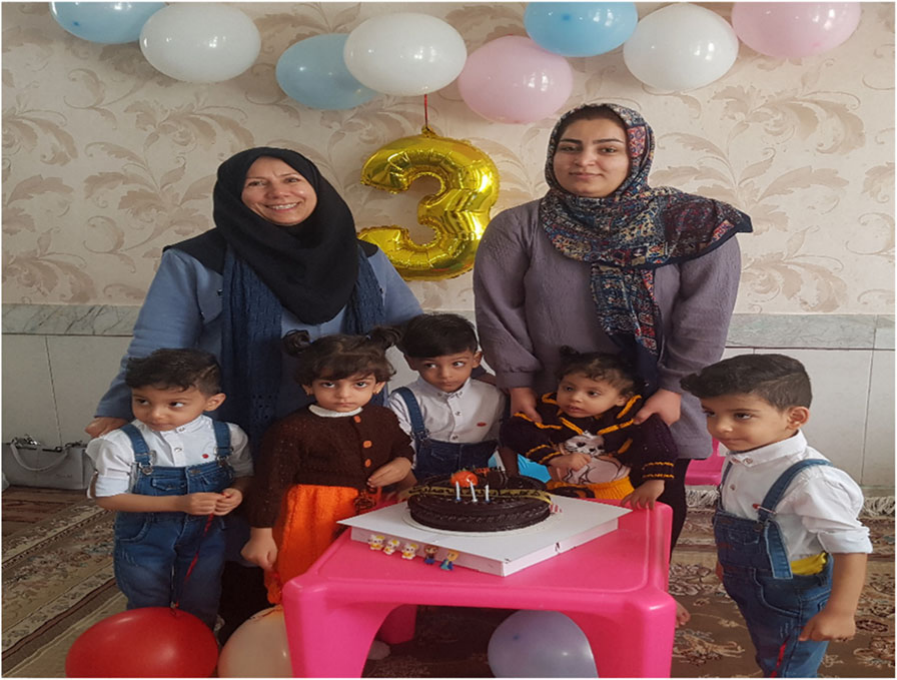
Picture 4: quintuplet at age of three (Namnabati, Isfahan University of Medical Sciences, Isfahan, Iran.2020)

According to the standard growth chart, physical and developmental growth of the multiple-birth infants is a delaying growth during the first 18 months up to the normal curve (except one of them) as described below. The quintuplets were born at 29th gestational week in a hospital in Isfahan in 2018. Two of them were girls and three others were boys weighing 1300, 1100, 1000, 1120, and 1130 g respectively, and were in need of artificial ventilation and surfactant. They were discharged with gavage tubes 26 days after the delivery. Two of them were re-hospitalized for apnea and discharged again after two weeks. The physician ordered the discharge accompanied by pulse-oximeter and oxygen cylinder enabling the parents to monitor and regulate the infants’ oxygen saturation at home. The 24-years-old mother of quadruplets, under medical treatments, delivered her infants through cesarean at 32nd gestational week in a hospital in Isfahan in 2019. They weighed 1370, 1210, 1170, and 1530 g and all of them were discharged two weeks after the delivery. The first home care and visit was performed few weeks after the discharge. However, special cares were given either on site or through phone calls. The infants were under regular and periodic care and treatment and were followed up for developmental growth for two years. Both family and healthcare team tried to maintain physical growth, weight gain, and some of the physiological indicators at an optimal level. According to the growth curve, the weight gains of the infants were between 40 to 50% in the growth chart. The average weight of the infants was 1130 g at the delivery time, 1930 g after 40 days, 5412 g after 6 months, 7820 g after one year, 11,800 g at the age of 2, and 13,400 g at the age of 3 (Figs. 2, 3, 4, 5).

A delay in developmental trend of a preterm infant is predictable for the first two years of the life. These infants were examined by neonatologist and were supervised in clinic for developmental tests like Bayley Scales of Infant Development. They were also followed up for screening of their eyes, kidney and bone. All of them were in a healthy condition except one girl who had a retard development. She was rehabilitated 2–3 times per week.

### Home visit and home care

To do special cares, home visits and home cares began a few weeks after the births. The infants were examined and cared by the NICU nurses 4–5 h every day during the first week. A speech therapist tried to improve oral feeding through Beckman exercises for the retarded girl. In addition, follow-up interventions were conducted by phone calls. Other medical members such as neonatologist, neurologist, and ophthalmologists visited quintuplet as needed during these two years.

The most interesting and important nursing care for the quintuplet was the infants’ feeding management. During the first days, five caregivers were needed for each meal. Every feeding round took about two hours to use 25 to 30 ml of formula or breast milking, using gastric tube, and cups, breast feeding and nasal feeding by the grandmothers, two nurses, and the mother. The mother, grandmother, relatives and the caregivers were completely exhausted from the feeding and interestingly it was the time for the next meal. Gradually, mother and grandmother learned how to schedule and program the infants’ feeding.

### Development of care plan

A care plan was developed based on the nursing process to manage the comprehensive care. The nursing process consisted of four phases of nursing diagnosis, goal, interventions (training), and evaluation. An example of a nursing diagnosis is shown in Table 1.

**Table 1:** Multiple-birth infants Care Plan

Nursing Diagnosis	Goal	Interventions	Evaluation
- Ineffective breast feeding related to prematurity and poor sucking (infants did not have adequate oral stimulation for about 30 days)	-To enable the mother to breastfeed for about 20 min for at least 1–2 times a day -The infant’s effective breastfeeding 1–3 times with proper breast placement in the mouth …	1- Mother’s training on the importance of breastfeeding 2. Breastfeeding training with tube feeding 3. Teaching the mother about the different breastfeeding techniques and choosing the most appropriate ones …	Mothers’ ability to breastfeed her baby at least twice. - The infants were fed and monitored through tube feeding, cups, connectors and bottles.

### Socio-economic support

Financial issue is the most important challenge for the parents of Multiple-birth infants. They have to provide clothing, diapers, and formula for four or five babies, requiring the support of the government and benefactors to deal with their needs. In the present study some of these needs were met through State Welfare Organization of Iran and some others by the benefactors, and Isfahan University of medical Sciences. But, as the infants grew older the formula was not suitable enough to meet their needs leading to gastroenteritis disorder. Therefore, the families had to procure their needs from pharmacies at a relatively higher price.

Maintaining the health of the infants imposed high charges on the health system, a part of which was compensated by Isfahan University of Medical Sciences affiliated departments or by the Welfare Organization. Some of the authorities also helped the families in cash in the form of present credit cards during their occasional visits.

Living place of the infants was another issue that needed to be modified. Physical limitations, lack of separate room and mattress, exposure to intensive light and noise, existence of numerous steps for going to the second floor, and substandard home design were all noticeable issues resulting in the infants’ sleep disturbance and discomfort. Taking into account the developmental care standard requirements, first the parents were familiarized with suitable and standard environment and, then, the place was made relatively suitable when some peripheral corrections and modifications were implemented. Later, spending the money donated by charities, a big bed was purchased for the quadruplet and a first floor flat was rented.

Simultaneous care of four and five infants requires the cooperation of the whole family members or even hiring caregivers. Thanks God, in the Iranian culture, grandmothers are in many cases available to help eagerly. But sometimes they, themselves, need some helps for personal problems such as hypertension, arthritis, and diabetes. Therefore, a skilled caregiver was employed and paid for by the representative of a donor. The hired caregiver was then trained about nutrition, resuscitation and health care; but she resigned after few months objecting to high work load and leaving the family with the previous difficulties. The fathers of the two groups had low incomes from temporary jobs. However, because they were needed to help and cooperate in care of the infants they missed their jobs leading to more financial problems. We appreciate one of the benefactors who hired them.

### Coronavirus nightmare

Attacking the people worldwide, COVID-19 has become a big problem for both people and health systems. There were 4 or 5 babies in a room. To avoid coronavirus neither could the infants be taken out of their room nor could the caregiver enter the room easily. In an interview the mother said:

*“Holding four infants in a room in order to avoid any contact from the others and not letting even the close relatives enter the room to stay away from Corona has become a nightmare to me … For this, I could not go to health clinics for vaccination like before*”. These limitations had made a nightmare out of corona for these families. (Table 2).

**Table 2:** of Challenges Strategies of multiple-birth infants

Challenges	Strategies
**1. Sterility and Infertility period**	1. Informing young couples about pregnancy and the consequences of taking ovulation-stimulating drugs 2.Comprehensive awareness about the consequences of taking stimulant and multiparous drugs 3. Providing the couples with the opportunity to use appropriate methods for pregnancy 4. Psychosocial support of young couples for fear of childlessness 5. Informing the couples’ families and preventing infertility-related cultural labels
**2. Transition from Intrauterine to NICU**	1. Providing facilities for the presence of mother in the NICU from the time of admission 2. Preventing the separation of the mother from the infant 3. Empowering and training mothers and key family members in the hospital 4. Distributing care duties among the family members 5. Psychological and family support for the parents
**3. Discharge Process**	1. Preparing the parents, hospitals, and charities for the process 2.Teaching how to care for the babies at home during pregnancy and hospitalization 3. Preparing the parents for early discharge 4. distributing the duties between the parents and preparing the relatives for support
**4. Physical-development status**	1. The need for a regular plan for periodic care and examinations 2. The need to train and equip their home if necessary 3. Necessity of specialist examination and precise treatment of preterm infants by specialists 4. Mental support for the mother and family
**5.Home care and home visit Challenges**	1. Familiarizing the nurses about home visit and hospital care 2. Assign a nurse responsible for encouraging the mother to continue breastfeeding 3. Enabling and empowering the mothers and key family members to take care of babies 4. Distributing care duties among the family members 5. Psychological support for the mother and the family 6- Two fixed caregivers in two shifts to assist the mother 7. Promoting a home-based care culture in the society
**6. Developing care plan**	1. Completion of care plan by all members of the health team 2. The need to identify the needs of the multiple-birth infants 3. Providing a comprehensive care package for the multiple-birth infants 4. The need to form a comprehensive child care team
**7.Socio-Economic Support**	1. Supportive organizations to hire the jobless fathers 2. Support of family and friends 3. Supply of formula and supplements in the infancy period 4.Timely and appropriate support by the Welfare Organization and other organs 5. Spreading the culture of helping others
**8.Coronavirus nightmare**	1. Giving information about risk factors and signs 2. Emphasizing that parents stay home with their quintuplet or quadruplet 3.Teaching the parents about hand washing and wearing mask 4. Providing the families with some entertainments 5. Asking health ministry for infants at home vaccination.

## Discussion

According to the results of the present study, a multifetal delivery imposes many problems on the family and the health system. While families are waiting for one more member to be added to their family, four or five infants may lead to a family crisis. Medical records of the couples in our study indicated that they had used inductive fertility drugs during their youth. Therefore, we reminded physicians and families that abortion is forbidden in accordance with religious rules and laws and has some consequent punishments. However, some malformations that may lead to a loss of life are among the exceptions where an abortion is permitted and such an abortion can be compensated based on religious practical instructions. One of several fetuses may also be aborted independent of others. To receive the permission for reducing the fetus, referring to applied religious documents can be useful. Artificial fetus should be treated differently [15].

Hospitalization was considered as a transition from intrauterine world to the NICU. Therefore, as it is indicated in Table 2, this is a suitable time to prevent mother–infant separation and enable the mother to care for her infant from the very beginning of hospitalization by emotional and psychological supports [16].

The process of discharge program is another challenge that needs policy-making and planning shown in Table 2. The package of home visit program was developed in Iran and executed as pilot in Isfahan University of Medical Sciences supported by infant health organization affiliated to the Ministry of Health. The package was later executed in Tabriz, Tehran, and Gorgan cities during the following years. According to the package, coordination for home care is triggered since hospitalization. Some care activities are performed at home when the parents’ consent is received [17, 18]. However, it should be noted that an especial package needs to be developed for the multifetal deliveries with respect to their needs in order to minimize their problems.

Preterm delivery has inevitable consequences such as nutrition disorders and infection. Families may also encounter some problems like audible, speech, and different ratings of vision in their infants. In a study conducted in the infant wards of Tehran University of Medical Sciences, it was shown that 12% of 99 multifetal infant samples of the study aging 4–9 weeks suffered retinopathy [19]. Therefore, based on the considerable points of Table 2, proper decisions should be made in this regard.

The contents of home visit and care are adopted from the needs of the society to improve the quality of healthcare. Advancement of the home care industry has recently conquered the limits of traditional care giving. Also, the costs of health services and advancement of medical and communication technology have been controlled, resulting in discharge of the infant from the hospital “quicker and sicker”.

Training the families about the health of their infant and home care of elders was established in the US in 1800; thereafter, insurance companies covered up these services. Home care programs were triggered in Canada more than 35 years ago. It is emphasized in these programs that short time hospitalization is achievable when the caregiver assures the patient for suitable and sufficient follow up. Several studies, conducted in several states, indicated that the duration of hospitalization is not enough to meet the needs of the infants and their families, as further follow-up is needed by the caregiver (18–19). Therefore, based on the considerable items of Table 2, it is suggested that a home care program be designed with respect to their needs.

Our experience with the table resulted in planning a separate care program based on nursing process last year. The barriers of the care plan were reviewed and listed with respect to the infants’ needs after the first visit. The plan is used to improve, maintain, and rehabilitate family and infants’ health [19].

Socio-economic support is an inevitable need of multifetal families. To our knowledge, they are mostly from low and average income families of the society. Financial shortcomings, missing the job by either mother or father, and loneliness in care giving demand for social supports. When population growth is the government’s strategy, financial support of the population is seriously and inevitably required [19]. Additionally, with respect to the items of Table 2, suitable and sufficient supports from the Welfare Organization and other institutes is needed to procure food, diapers, clothing, suitable residence and appropriate job for the jobless fathers of the family. Social supports could also yield better growth and developmental condition if a caregiver could be hired to help the family. Could the parents know about the type and amount of the supports, their expectations would be balanced and they would be ensured about the future of their infants [17].

Generally, to ensure survival and suitable growth and development of multiples, a home care program can be planed based on the needs of infants from the prenatal to infancy period including support for their parents during the hospitalization, the first 3 h after hospital discharge, 2–3 days later and, then, based on a regular schedule the coming years until school age and more.

Although a home visit program for parents with preterm infants has been launched by the Neonatal Health Department of the Ministry of Health of Iran [17], the program has limitations with regard to regular visits for triplets, quadruplets, and quintuplets. Therefore, a program must be developed for multiple infants based on the result of this study. Furthermore, as this was a qualitative case study that explored a phenomenon within some particular context through various data sources, we cannot generalize the result of study.

## Conclusion

This study provided a comprehensive and complete picture of the challenges of the multiple-birth infants and and the ways of coping with them. The care plan can facilitate the growth and development of the premature infants. Being with these families for two years empowered the parents and made them enable to adapt to special situations.

With regard to the increase of multifetal deliveries, this study showed that the needs of the families should be specified and an inclusive and comprehensive care program should be developed based on the existing experiences and in cooperation with related organizations such as University of Medical Science, Governmental Treatment Centers, State Welfare Organization, and other benefactors.

## Data Availability

The data of the current study is available from the corresponding author (MN) on request.

## Abbreviation

NICU: Neonatal Intensive Care Unit
